# Is JAK effective in treating recurrent SAPHO syndrome? TwHF might be a good choice

**DOI:** 10.1097/MD.0000000000038848

**Published:** 2024-07-19

**Authors:** Qiong Sun, Qinchen Gu, Haixu Jiang, Weizhong Li, Zhimin Lin, Chen Li, Zhenhua Ying

**Affiliations:** a The Second College of Clinical Medicine, Zhejiang Chinese Medical University, Zhejiang 310014, China; b Department of Rheumatology and Immunology, Center for General Practice Medicine, Zhejiang Provincial People’s Hospital (Affiliated People’s Hospital, Hangzhou Medical College), Zhejiang 310014, China; c School of Chinese Materia, Beijing University of Chinese Medicine, Beijing 102488, China; d Department of Rheumatology, Fangshan Hospital, Beijing University of Chinese Medicine, Beijing 102401, China; e Third Affiliated Hospital, Beijing University of Chinese Medicine, Beijing 100700, China; f Institute of Rheumatology and Immunology, Hangzhou Medical College, Zhejiang 310014, China; g Zhejiang Provincial Key Laboratory of Traditional Chinese Medicine Cultivation for Arthritis Diagnosis and Treatment, Zhejiang 310014, China

**Keywords:** JAKi, SAPHO syndrome, TwHF

## Abstract

**Background:**

Recently, JAKi has also been widely proved to be an effective alternative to conventional treatment for Synovitis acne pustulosis–hyperostosis–osteitis (SAPHO) cases, after failure of multiple drugs including those described above. But what to do when all these treatments fail? We report a case of remission from Tripterygium wilfordii Hook (TwHF) treatment.

**Methods:**

The patient was treated with nonsteroidal anti-inflammatory drugs, oral prednisone, minocycline, bisphosphonate injection, etanercept, and tofacitinib, but the symptoms did not change significantly. Treatment with TwHF (1.0 mg/kg/day, patient weight 60 kg) was started for 24 weeks.

**Results:**

After 50 months of unsatisfactory treatment, this patient was finally treated with herbal TwHF, and after 6 months of treatment, the patient’s magnetic resonance imaging and inflammatory indexes were significantly improved, indicating that the disease had been better controlled.

**Conclusion:**

In this study, TwHF was successful in treating a patient with refractory SAPHO syndrome who was refractory to multiple Western medications without significant adverse effects or toxicities, but further follow-up is needed to determine long-term efficacy. More case reports as well as clinical trials are still needed to confirm whether TwHF can effectively treat refractory SAPHO syndrome.

## 1. Introduction

Synovitis acne pustulosis–hyperostosis–osteitis (SAPHO) syndrome is a rare disease with dominant osteoarticular and dermatological manifestations. Typical osteoarticular signs include osteitis, hyperostosis, synovitis and arthropathy, and skin lesions include palmoplantar pustulosis and severe acne.^[1]^ Currently, in terms of clinical management, conventional therapy includes NSAIDs, antibiotics, glucocorticoids, anti-TNF-α agents and bisphosphonates. Nonsteroidal anti-inflammatory drugs (NSAIDs), antibiotics, and glucocorticoids, alone or in combination, are the first-line treatment for the majority of patients (approximately 37%), but are effective in only about 20% of cases. Bisphosphonates improved osteoarticular symptoms more significantly in patients with SAPHO syndrome (about 22%). Whereas in case of failure of conventional treatment, biologics became the treatment of choice, with TNFi being the preferred choice, with significantly different efficacy rates reported in different studies (25%–90%), unsatisfactory improvement of skin symptoms was followed by switching to treatment with targeted IL-17/IL-23 biologics (75%) or JAK inhibitors (60%–87%).^[2,3]^ Recently, JAKis have also been widely proven to be effective alternatives to conventional treatments for SAPHO syndrome patients after the failure of multiple drugs, including those described above.^[4]^ However, what to do when all these treatments fail? We report a case of remission from Tripterygium wilfordii hook f (TwHF) treatment.

## 2. Case presentation

**Figure 1.**
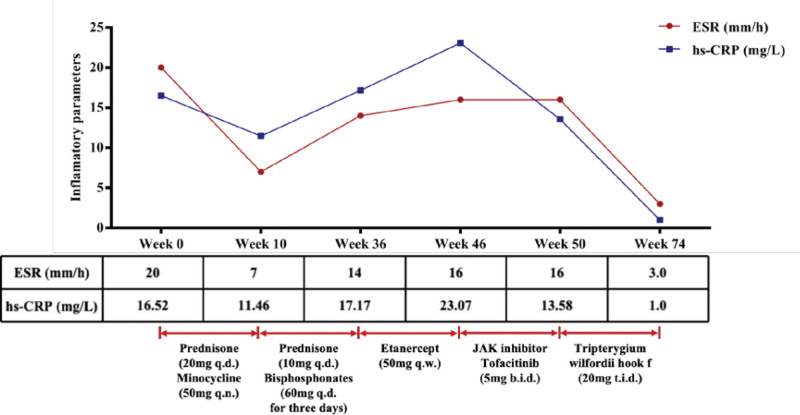
Dosage, frequency, and duration of different treatments after diagnosis. NSAIDs were used as needed. Before the diagnosis, NSAIDs and oral prednisone were given to reduce symptoms. The dose of prednisone after diagnosis was gradually reduced to 5 mg q.d. at month 8. ESR, reference range 0 to 20 mm/h; hsCRP, reference range 0 to 3.00 mg/L. ESR = sedimentation rate, hsCRP = augmented hypersensitivity C-reactive protein. NSAIDs = nonsteroidal anti-inflammatory drugs; q.d.= once a day; b.i.d. = twice per day; t.i.d.= 3 times per day.

**Figure 2.**
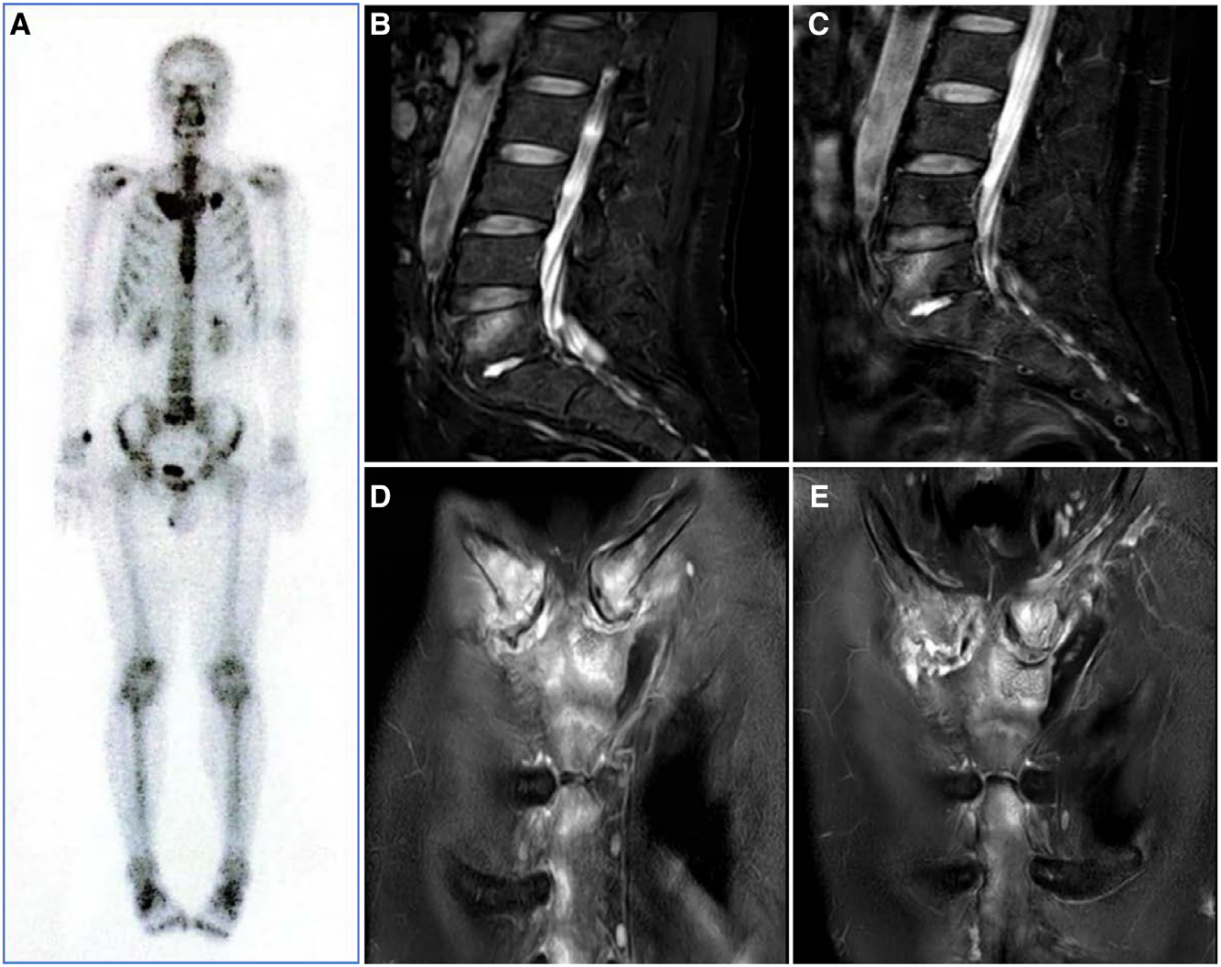
(A–E): Whole-body bone scan and magnetic resonance imaging of the whole spine of a 46-yr-old male who presented with the syndrome at week 0. Whole-body bone scan revealed good uptake and clear visualization of bones throughout the body, with areas of increased radioactivity uptake observed in the bilateral sternoclavicular joints and adjacent sternums, clavicles, lower sternums, right 8th rib proximal to the costovertebral joints, and the edges of the L5 and S1 vertebrae, while the remaining bones did not show any obvious abnormal areas of increased or decreased radioactivity. Sternoclavicular joint and Lumbar MRI suggested: Bilateral sternoclavicular and lumbar 5 vertebral bone marrow edema was less extensive and less intense than before.

A 46-year-old man suffering from therapy-refractory SAPHO syndrome for 5 years characterized by chest, joint and lumbosacral pain along with palmoplantar pustulosis. Whole-body bone scintigraphy revealed areas of increased radiolucent uptake visible bilaterally at the sternoclavicular joints and adjacent sternum, clavicle, lower sternum, and right 8th rib proximal to the costovertebral joints and at the margins of the L5 and S1 vertebrae (Fig. 1). Previous treatments with NSAIDs, oral prednisone (20 mg/d) and minocycline all failed to improve symptoms dramatically. Prednisone was then reduced by 2.5 mg/d at 2-week intervals, and skin abnormalities and pain in bone structures and adjacent tissues recurred when the dose increased to 10 mg/d. The patient’s bone pain was significantly reduced after the start of treatment with intravenous bisphosphonates (60 mg/d for 3 days), which were used at 3-month intervals, but symptoms recurred after 2 doses. Symptoms recurred after 10 weeks of etanercept (50 mg, qw) treatment. The patient was subsequently treated with tofacitinib (5 mg, twice/d) for 1 month, but the disease remained unsatisfactorily controlled.We started treatment with TwHF (1.0 mg/kg/d, the patient weighed 60 kg), the patient’s symptoms improved significantly after 24 weeks of treatment, and a repeat MRI showed marked improvement. At the 24th week of treatment, the ESR decreased to 3.0, and the CRP decreased to 1.0. Sternoclavicular joint and Lumbar MRI suggested: Bilateral sternoclavicular and lumbar 5 vertebral bone marrow edema was less extensive and less intense than before (Fig. 2B–E).

## 3. Discussion

Treatment of SAPHO syndrome is currently characterized by significant clinical heterogeneity. In our patient, the disease had persisted for a long time. After previous and subsequent treatment with cortisol, bisphosphonates, and methotrexate with etanercept, the symptoms still recurred, and the patient was considered refractory SAPHO syndrome. We then attempted treatment with a JAK inhibitor for 1 month, but disease control was still unsatisfactory. Based on previous therapeutic experience and corroborating results from relevant clinical trials, we believe that Tripterygium wilfordii hook f (TwHF) is effective in the treatment of patients with refractory SAPHO syndrome. TwHF, also known as Lei Gong Teng, is a Chinese herbal extract that has been widely used for treating inflammatory and autoimmune disorders in China because of its low cost. It has been shown to be effective in the treatment of active RA and psoriasis in several randomized controlled trials without a significant increase in adverse events.^[5,6]^ With respect to potential predictors, TwHF therapy has better outcomes than MTX therapy in patients with RA.^[7,8]^ The anti-inflammatory and immunosuppressive effects of TwHF are related to the regulation of inflammatory cytokines and the activities of T cells and dendritic cells. Triptriolide (a component of TwHF) is the main bioactive substance of TwHF and has been shown to have anti-inflammatory effects in different animal models and preclinical studies.^[9]^ Mechanistically, triptriolide significantly inhibits the number of Th17 cells, resulting in decreased mRNA expression of IL-17A, IL-22, and TNF-α.^[10]^ It can also effectively decrease oxidative stress responses and the production of inflammatory cytokines by regulating the activity of the nuclear factor erythroid 2-related factor 2 (Nrf2) transcription factor and cross-talk with NF-κB signaling pathways.^[7,11]^ In a single-center study evaluating the efficacy and safety of TwHF for the treatment of SAPHO syndrome, TwHF was found to be fast-acting, efficacious, and safe for the treatment of SAPHO syndrome.^[12]^

## 4. Conclusion

After 50 months of unsatisfactory treatment, this patient was finally treated with herbal TwHF, and after 6 months of treatment, the patient’s magnetic resonance imaging and inflammatory indices significantly improved, indicating that the disease had been better controlled. In this study, TwHF was successful in treating a patient with refractory SAPHO syndrome who was refractory to multiple Western medications without significant adverse effects or toxicities, but further follow-up is needed to determine its long-term efficacy. However, more case reports and clinical trials are needed to confirm whether TwHF can effectively treat refractory SAPHO syndrome.

## Acknowledgments

The authors are grateful to all participants for their participation in this paper.

## Author contributions

**Conceptualization:** Qiong Sun, Qinchen Gu, Haixu Jiang, Weizhong Li, Chen Li, Zhenhua Ying.

**Writing – original draft:** Qiong Sun, Qinchen Gu.

**Methodology:** Haixu Jiang, Zhimin Lin.

**Data curation:** Weizhong Li.

**Software:** Zhimin Lin.

**Funding acquisition:** Chen Li, Zhenhua Ying.

**Writing – review & editing:** Zhenhua Ying.
